# Supplementary material for: Evolution of Responses to COVID-19 and Epidemiological Characteristics in South Korea
Source: Int J Environ Res Public Health. 2022 Mar 29;19(7):4056. doi: 10.3390/ijerph19074056 (PMC8997838; doi:10.3390/ijerph19074056)
Supplement: Supplementary file 1 [file ijerph-19-04056-s001.zip › ijerph-1623326-supplementary.pdf]

International Journal of
Environmental Research and Public Health

Table S1. Administrative social distancing measures in South Korea from March 2020 to July 2021 retrieved from KDCA COVID-19 press release.

Date	Administrative measures	Symbol	Period
22 MAR 2020	Commencement of social distancing program	E1	P1
6 MAY	Relaxed to "social distancing in life"	R1	P2
16 AUG	Upgrade to Level 2 in Seoul metropolitan area (SMA)	E2	P3
23 AUG	Nationwide expansion of Level 2		
30 AUG	Upgrade to Level 2.5 in SMA	E3	
14 SEP	Downgrade to Level 2 in SMA.	R2	
12 OCT	Downgrade to Level 1 nationwide except some regions in SMA	R3	P4
7 NOV	Nationwide expansion of Level 1	R4	
19 NOV	Upgrade to Level 1.5 in SMA and some areas of Gangwon	E4	
23 NOV	Upgrade to Level 2 in SMA		
1 DEC	Upgrade to Level 1.5 nationwide outside SMA		
8 DEC	Upgrade to Level 2.5 in SMA and Level 2 nationwide outside SMA	E5	
28 DEC	Stronger Level 2.5 in SMA and stronger Level 2 nationwide outside SMA	E6	
15 FEB 2021	Downgrade to Level 2 in SMA and Level 1.5 nationwide outside SMA	R5	
26 FEB	Commencement of vaccination against COVID-19		P5
12 JUL	Upgrade to Level 4 (the highest level) in SMA	E7	P6
15 JUL	Upgrade to Level 2 nationwide except SMA, Sejong, Jeonbuk, Jeonnam, and Gyeongbuk		

Table S2. Linear regressions of confirmed cases vs unlinked and asymptomatic cases.

Type	Coefficients	P1	P2	P3	P4	P5	P6
Unlinked	Slope ($\times 10^{-4}$)	11.9	60.5	15.0	1.45	3.90	-0.20
	Pearson	0.557	0.376	0.668	0.386	0.463	-0.113
	p-value	0.000	0.001	0.000	0.000	0.000	0.365
Asymptomatic	Slope ($\times 10^{-4}$)	0.41	28.7	2.84	0.54	-0.51	-0.46
	Pearson	0.024	0.255	0.256	0.325	-0.137	-0.104
	p-value	0.809	0.026	0.015	0.000	0.112	0.407

Table S3. Regional shares of confirmed cases relative to population size with abbreviations Seoul (SL), Busan (BS), Daegu (DG), Incheon (IC), Gwangju (GJ), Daejeon (DJ), Ulsan (UL), Sejong (SJ), Gyeonggi (GG), Gangwon (GW), Chungbuk (CB), Chungnam (CN), Jeonbuk (JB), Jeonnam (JN), Gyeongbuk (GB), Gyeongnam (GN), and Jeju (JJ).

Period		SL	BS	DG	IC	GJ	DJ	UL	SJ	GG	GW	CB	CN	JB	JN	GB	GN	JJ
1	Unlinked	-16.0	-5.6	72.4	-5.4	-2.8	-2.6	-2.0	-0.3	-21.8	-2.6	-2.8	-3.7	-3.4	-3.5	7.3	-5.8	-1.3
	Linked	-9.6	-4.8	29.7	-3.8	-1.8	-2.3	-1.0	0.0	-10.3	-2.6	-1.7	2.4	-3.3	-3.3	16.5	-2.9	-1.2
2	Unlinked	23.4	-6.3	-1.1	2.0	4.5	2.2	-2.1	-0.7	3.5	-2.4	-2.4	-2.8	-3.3	-3.2	-4.2	-6.1	-1.2
	Linked	13.9	-6.2	-3.2	10.6	4.2	3.4	-2.2	-0.2	3.7	-2.4	-2.3	-2.3	-3.0	-2.8	-4.0	-5.9	-1.3
3	Unlinked	21.0	-3.9	-2.6	1.2	0.4	-1.4	-1.4	-0.5	7.5	-1.4	-2.5	-1.5	-2.8	-1.9	-4.0	-5.2	-1.0
	Linked	18.6	-2.4	-3.5	-0.8	0.3	0.7	-1.3	-0.5	7.5	-1.4	-1.8	-1.0	-2.3	-2.8	-3.0	-5.5	-0.8
4	Unlinked	19.6	-3.6	-2.8	-1.0	-2.2	-1.7	-1.4	-0.4	9.7	-0.1	-0.9	-1.2	-2.9	-3.1	-2.5	-4.5	-1.0
	Linked	13.6	-1.4	-2.1	1.2	0.8	-1.4	-0.6	-0.5	0.8	-0.7	-0.4	-0.9	-1.3	-2.0	-2.3	-2.7	-0.2
5	Unlinked	18.7	-2.0	-3.6	-1.1	-2.0	-0.8	0.1	-0.2	7.3	0.3	-1.1	-2.0	-2.4	-3.0	-3.6	-3.7	-0.7
	Linked	10.5	-2.3	0.5	-3.1	-0.9	0.0	-0.6	-0.2	3.9	-2.0	-0.8	-2.1	-1.0	-1.7	-1.8	0.2	0.2
6	Unlinked	20.2	-4.4	-3.0	0.1	-2.1	-0.6	-1.5	-0.4	10.1	-0.7	-1.8	-1.6	-2.8	-3.1	-4.0	-4.5	-0.7
	Linked	-12.8	7.5	3.9	-2.1	0.7	3.3	2.7	0.2	-15.5	-2.0	1.1	3.3	0.1	-0.7	1.2	7.4	2.0

Table S4. Shares of confirmed cases normalized by the population size in each age bracket after Shincheonji church mass infection in P1 and 8/15 rally in P3.

Period	Type	< 20	20–29	30–39	40–49	50-59	60–69	70-79	> 79
Shincheonji	Unlinked	-0.106	0.257	-0.032	-0.033	0.008	-0.027	-0.036	-0.030
	Linked	-0.054	0.079	-0.011	-0.026	0.047	-0.006	-0.018	-0.010
8/15 rally	Unlinked	-0.095	-0.053	-0.056	-0.040	0.044	0.148	0.056	-0.004
	Linked	-0.058	-0.017	-0.036	-0.065	0.028	0.077	0.056	0.015

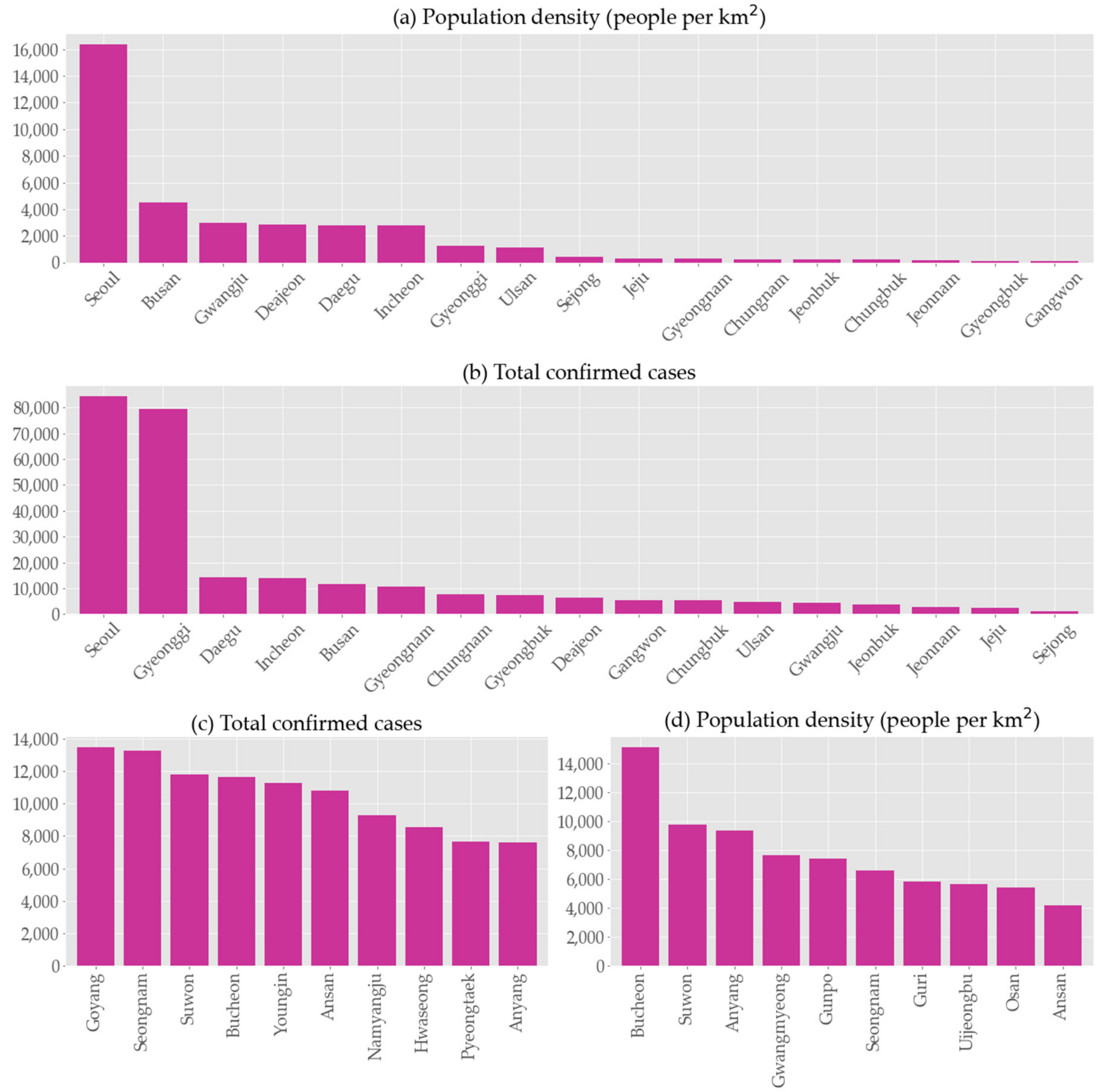

Figure S1. **(a)** Population density by region, **(b)** regional total numbers of confirmed cases by 16 SEP 2021, **(c)** regional total numbers of confirmed cases in Gyeonggi by DEC 2021, and **(d)** regional population density in Gyeonggi.

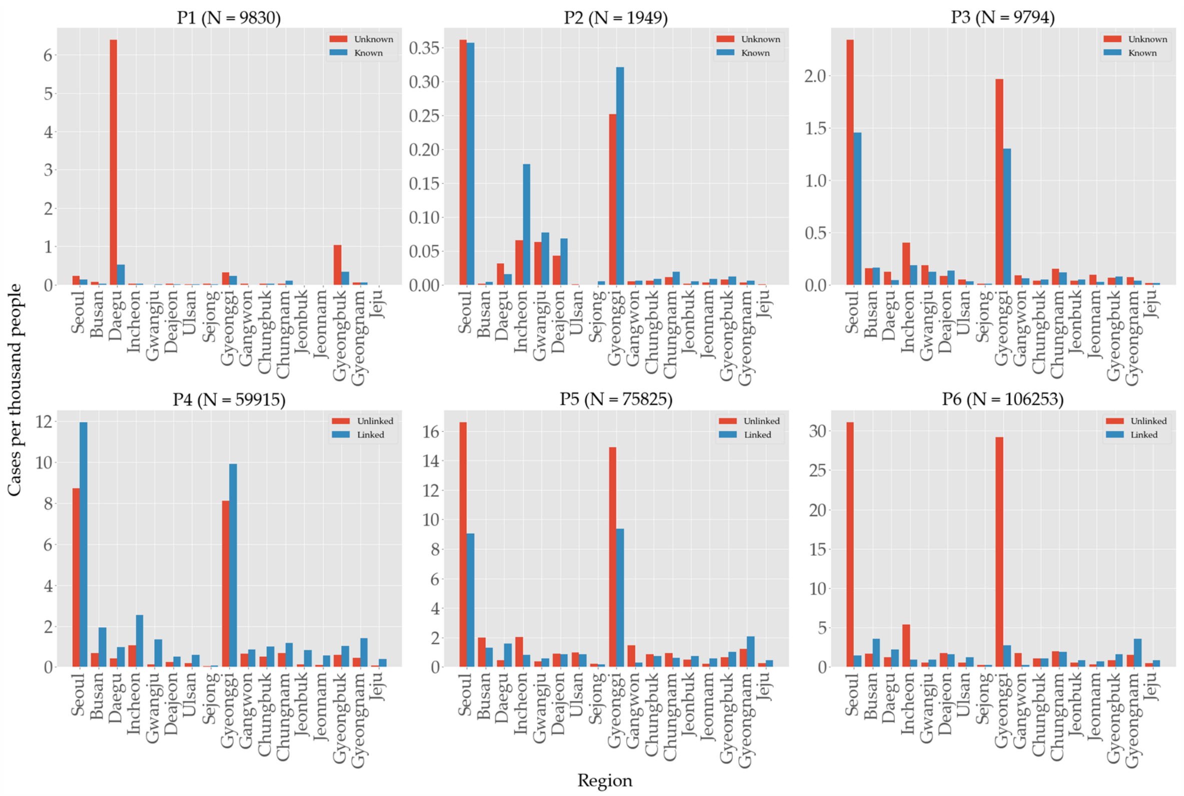

Figure S2. Regional numbers of infections per 1,000 people.

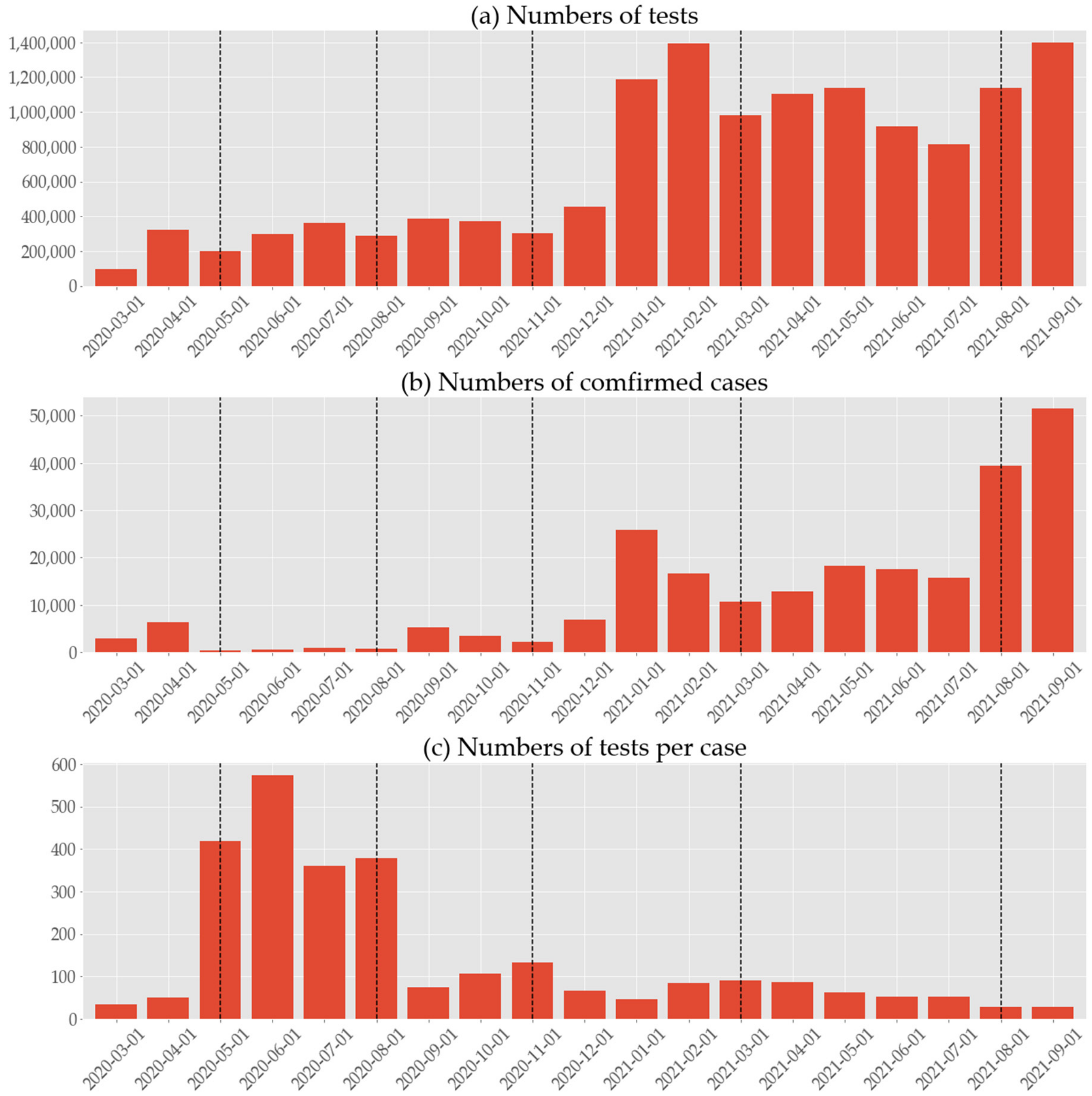

Figure S3. Monthly numbers of **(a)** tests, **(b)** cases, and **(c)** tests per case.

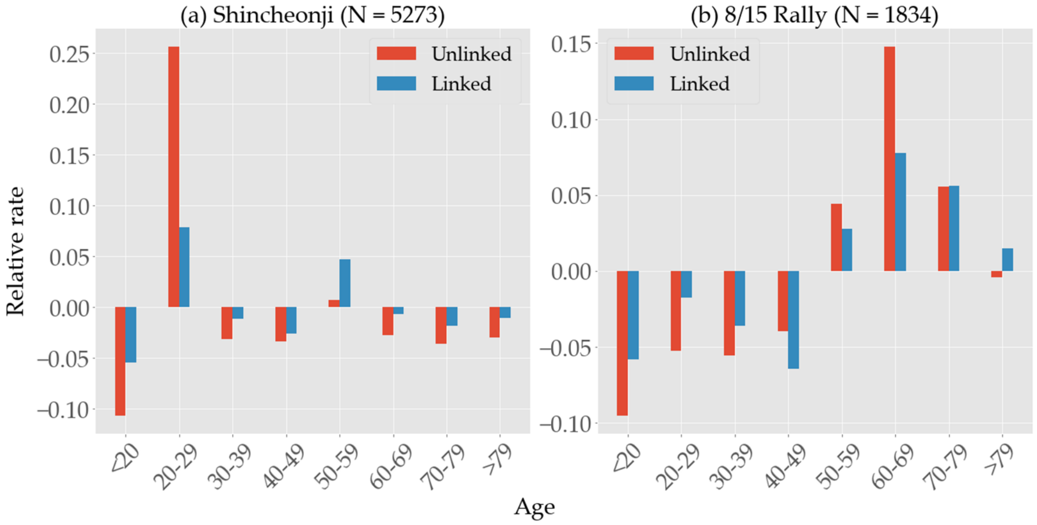

Figure S4. Shares of confirmed cases normalized by the population size in each age bracket **(a)** after Shincheonji church mass infection in P1 and **(b)** 8/15 rally in P3, contrasting two age groups severely affected by the incidents.

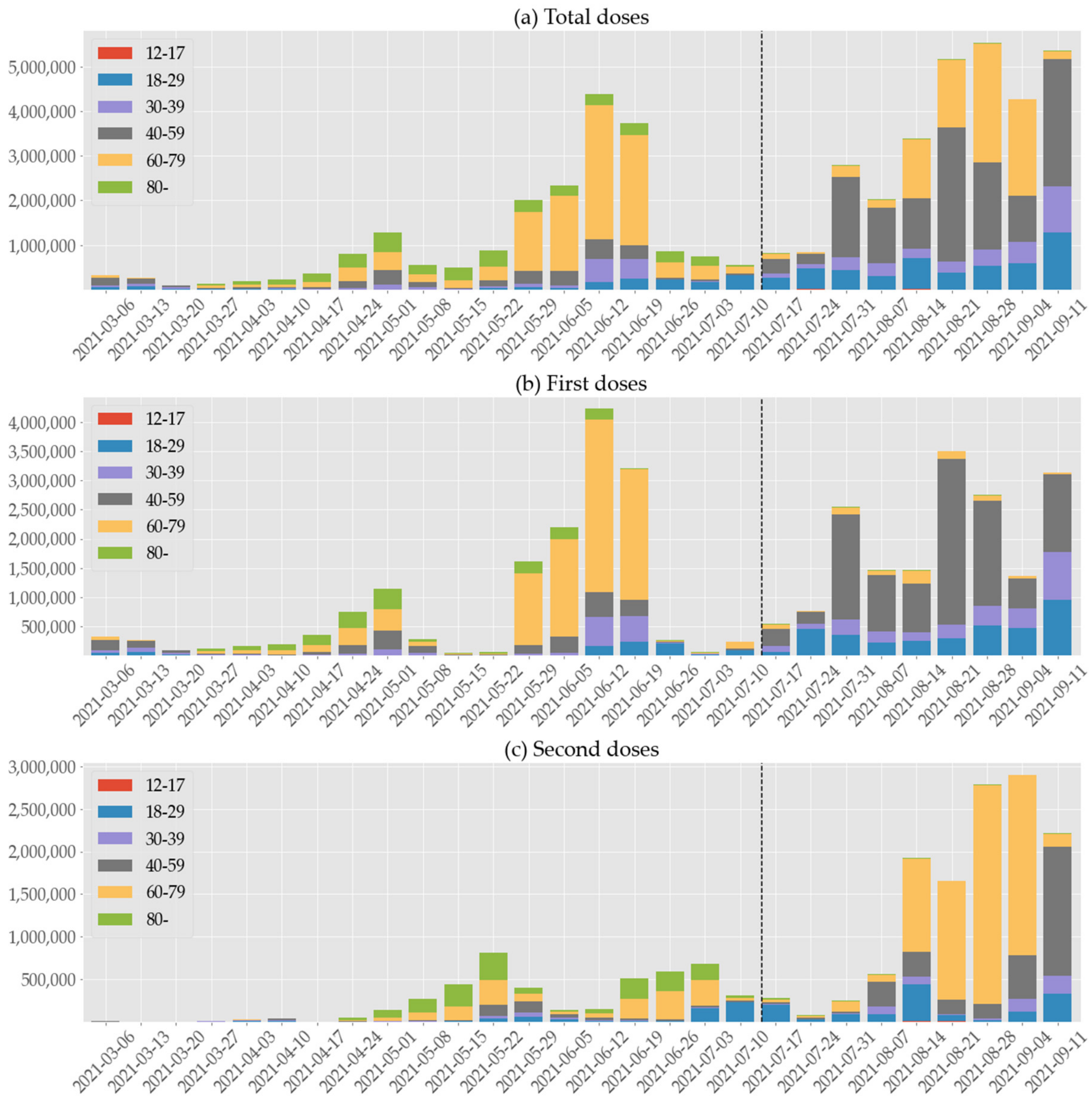

Figure S5. Weekly vaccine uptake in age groups **(a)** total doses, **(b)** first doses, and **(c)** second doses.

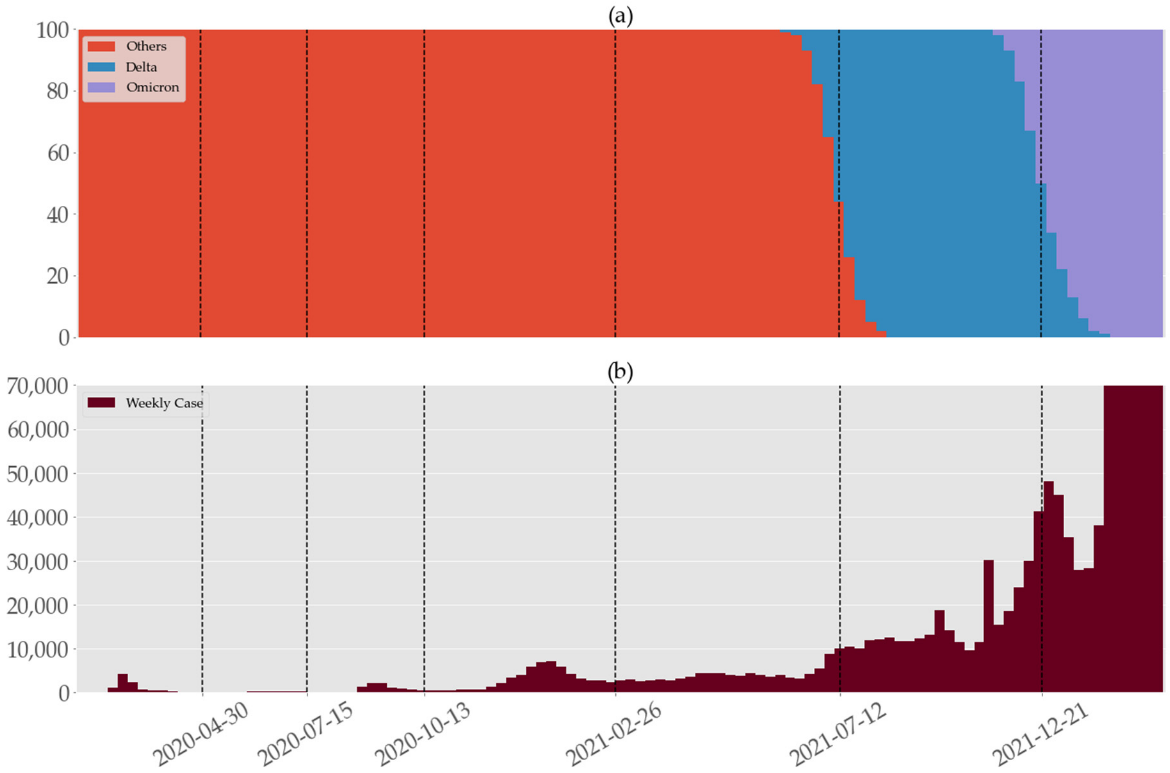

Figure S6. (a) Weekly percentage of variants of concern from 30 January 2020 to 27 February 2022. "Others" includes early mutations such as alpha and beta. The first five vertical lines delineate the 6 periods in the manuscript, while the last one marks the time when the omicron variant exceeds 50%. **(b)** Weekly numbers of confirmed cases where data exceeding 70,000 were truncated.
